# Melatonin-Regulated Chaperone Binding Protein Plays a Key Role in Cadmium Stress Tolerance in Rice, Revealed by the Functional Characterization of a Novel Serotonin *N*-Acetyltransferase 3 (*SNAT3*) in Rice

**DOI:** 10.3390/ijms25115952

**Published:** 2024-05-29

**Authors:** Hyoung-Yool Lee, Kyoungwhan Back

**Affiliations:** Department of Molecular Biotechnology, College of Agriculture and Life Sciences, Chonnam National University, Gwangju 61186, Republic of Korea; xanthine657@jnu.ac.kr

**Keywords:** binding proteins, chaperone, cadmium tolerance, melatonin, serotonin *N*-acetyltransferase, transgenic rice

## Abstract

The study of the mechanisms by which melatonin protects against cadmium (Cd) toxicity in plants is still in its infancy, particularly at the molecular level. In this study, the gene encoding a novel serotonin *N*-acetyltransferase 3 (*SNAT3*) in rice, a pivotal enzyme in the melatonin biosynthetic pathway, was cloned. Rice (*Oryza sativa*) *OsSNAT3* is the first identified plant ortholog of archaeon *Thermoplasma volcanium SNAT*. The purified recombinant OsSNAT3 catalyzed the conversion of serotonin and 5-methoxytryptamine to *N*-acetylserotonin and melatonin, respectively. The suppression of *OsSNAT3* by RNAi led to a decline in endogenous melatonin levels followed by a reduction in Cd tolerance in transgenic RNAi rice lines. In addition, the expression levels of genes encoding the endoplasmic reticulum (ER) chaperones *BiP3*, *BiP4*, and *BiP5* were much lower in RNAi lines than in the wild type. In transgenic rice plants overexpressing *OsSNAT3* (SNAT3-OE), however, melatonin levels were higher than in wild-type plants. SNAT3-OE plants also tolerated Cd stress, as indicated by seedling growth, malondialdehyde, and chlorophyll levels. *BiP4* expression was much higher in the SNAT3-OE lines than in the wild type. These results indicate that melatonin engineering could help crops withstand Cd stress, resulting in high yields in Cd-contaminated fields.

## 1. Introduction

Cadmium (Cd) is believed to act as one of the most harmful heavy metals for living organisms and is positioned 7th among the 20 most toxic metals; it is also a group 1 carcinogen [1]. In plants, Cd causes severe damage to growth and development, including by inhibiting photosynthesis [2,3], disrupting the ultrastructure of chloroplasts [4,5] and the endoplasmic reticulum (ER) [6], increasing the production of reactive oxygen species (ROS) [7], and disturbing cellular protein homeostasis [8]. To alleviate the adverse effects of Cd stress, plants have evolved a series of adaptative mechanisms against Cd toxicity. The first line of defense is the plant cell wall, which prevents Cd from entering cells by chelating it to cell wall components such as pectin and hemicellulose [9]. However, for Cd that invades inside cells, a number of defense responses are simultaneously induced to minimize Cd stress, such as Cd sequestration into vacuoles, Cd chelation by cytoplasmic organic acids or proteins, the enhanced production of antioxidant enzymes or other antioxidants, and the induction of heat shock proteins (HSPs) [5,8].

HSPs play a pivotal role in cellular protein homeostasis by acting as molecular chaperones of protein folding and hindering protein aggregation [10]. They are also induced in response to Cd, as well as other environmental stresses. In plants, there are five major classes of HSPs, identified based on their molecular masses: HSP60, HSP70, HSP90, HSP100, and small HSPs. HSP70 is highly conserved between bacteria and eukaryotes and is closely associated with the defense response in a wide range of plant species [11]. It maintains protein homeostasis by preventing the aggregation of stress-damaged proteins [12]. The rice genome contains 32 *HSP70* genes, whose protein products are localized in distinct subcellular compartments, including the cytoplasm, chloroplasts, and ER [12]. HSP70 proteins that reside in the ER are also referred to as binding proteins (BiPs). Like other HSP70 proteins, BiPs act as chaperones and interact with nascent immature proteins to facilitate their correct folding and assembly [13]. In the rice genome, there are at least five *BiP* genes, sharing >66% amino acid identity [14]. Among them, the expressions of *BiP4* and *BiP5* are positively correlated with the severity of ER stress [13], whereas *BiP1* expression plays a key role in the regulation of seed storage proteins [15]. While many environmental stresses, such as heat, salt, and drought, as well as heavy metal stress, evoke ER stress by causing dysfunctional protein folding [16,17,18], whether the amelioration of ER stress by *BiP* genes would improve plant growth and development remains elusive [18].

Melatonin is found in almost all living organisms, including animals, bacteria, archaea, and plants [19,20,21,22]. In animals, melatonin acts as a neurohormone, influencing circadian rhythms and seasonal reproduction [23]; other functions include energy metabolism, as well as anti-inflammatory, anti-cancer, and anti-aging effects [24]. In plants, however, melatonin does not function as a hormone, but rather as a signaling molecule, orchestrating a diverse array of physiological functions including growth and development, while also participating in defense responses against biotic and abiotic stresses [25] through protein quality control [26,27]. In plants, melatonin biosynthesis begins with the conversion of tryptophan into tryptamine in a reaction catalyzed by tryptophan decarboxylase, with intermediate serotonin—the last common substrate for melatonin biosynthesis in both animals and plants—produced through the enzymatic reaction of tryptamine 5-hydroxylase, located in the ER. Serotonin is converted into *N*-acetylserotonin or 5-methoxytryptamine by serotonin *N*-acetyltransferase (SNAT) or serotonin *O*-methyltransferase. The same enzymes catalyze the final steps that lead to melatonin biosynthesis [20]. Among these four enzymes, SNAT, as both the penultimate and final enzyme for melatonin biosynthesis depending on the substrate, has received considerable research attention due to its rate-limiting role in melatonin synthesis in animals and plants [19,28,29]. In plants, two *SNAT* isogenes, *SNAT1* and *SNAT2*, have been cloned and their respective proteins have been expressed in chloroplasts [30,31].

In this study, we cloned a third *SNAT* from rice (*Oryza sativa*), *OsSNAT3*, identified as an orthologous gene of the archaeon *Thermoplasma volcanium SNAT* [21]. In our experiments, we found that, in contrast to OsSNAT1 and OsSNAT2, OsSNAT3 is located in the cytoplasm. However, like *OsSNAT1* and *OsSNAT2*, the overexpression of *OsSNAT3* enhanced melatonin synthesis in transgenic rice, while its downregulation reduced it. It also conferred tolerance against Cd stress, while its downregulation increased the susceptibility of rice plants to Cd stress. These observations may be due to the differential expression of *BiP* genes. This is the first study to show that melatonin-regulated *BiP* regulation plays an important role in conferring Cd tolerance in rice, thus highlighting the close relationship between melatonin-induced *BiP* genes and Cd tolerance in plants.

## 2. Results

### 2.1. Rice (Oryza sativa) SNAT3 (OsSNAT3) Sequence Features, Bacterial Expression, and Purification of Recombinant OsSNAT3 Protein

**Figure 1:**
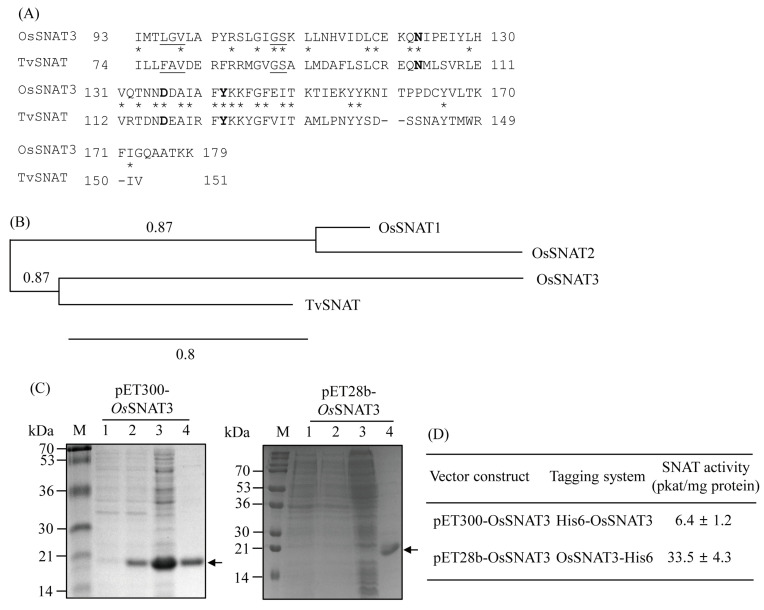
(**A**) Amino acid sequence alignment between archaeon TvSNAT and rice OsSNAT3. The conserved acetyl coenzyme A binding sites are underlined and key residues for SNAT activity are shown in bold. Stars indicate identical amino acids; dashes denote gaps. (**B**) Phylogenetic tree of *OsSNAT3* among multiple *SNAT* genes in rice. The scale bar represents 0.8 substitutions per site. GenBank accession numbers are NC_003413 (*TvSNAT*), AK059369 (*OsSNAT1*), AK068156 (*OsSNAT2*), and AK241100 (*OsSNAT3*). (**C**) Purification of His6-tagged OsSNAT3 proteins. *E. coli* BL21 (DE3) cells harboring pET300-OsSNAT3 and pET28b-OsSNAT3 plasmids were induced with isopropyl β-d-1-thiogalactopyranoside (IPTG) for 5 h at 28 °C. M, molecular mass standards. Lane 1, total proteins in 15 µL bacterial culture without IPTG; lane 2, total proteins in 15 µL bacterial culture with IPTG; lane 3, 30 µg soluble protein; lane 4, 5 µg affinity-chromatography-purified protein. (**D**) SNAT activity measured in purified N-terminal His6-tagged OsSNAT3 and purified C-terminal His6-tagged OsSNAT3. Protein samples were separated by SDS-PAGE on a 12% polyacrylamide gel and stained with Coomassie blue.

A BLAST search showed that the rice gene with the highest homology to the archaeon *Thermoplasma volcanium* SNAT (TvSNAT) [21] is OsSNAT3, previously annotated as rice *N*-alpha-acetyltransferase 50 (OsNaa50). OsSNAT3 had a 36.25% protein-level identity with TvSNAT in its C-terminal 80 amino acids (Figure 1A). A Conserved Domain Database (CDD) search [32] showed that OsSNAT3 has a conserved acetyl CoA binding site made up of L96, G97, V98, G108, and S109, and that it harbors key residues for SNAT catalysis, including N123, D136, and Y142 [33]. OsSNAT3 has a high amino acid sequence identity (51%) with human Naa50 [34], which also possesses SNAT activity [35]. In the rice genome, two SNAT isogenes, *SNAT1* and *SNAT2*, have been identified thus far. While both participate in melatonin biosynthesis, they differ in their downstream signaling responses in the brassinosteroid signaling pathway [20]. Phylogenetic analysis clearly indicated that *OsSNAT3* (or *OsNaa50*) is distantly related to the previously identified *SNAT* isogenes *OsSNAT1* and *OsSNAT2* (Figure 1B). To assess whether rice *OsSNAT3* harbors SNAT activity, recombinant OsSNAT3 was purified by expressing full-length *OsSNAT3* in *Escherichia coli*. As shown in Figure 1C, OsSNAT3 was expressed as a soluble protein and successfully purified on a Ni^2+^ affinity column.

### 2.2. Characterization of OsSNAT3 Enzyme Kinetics

**Figure 2:**
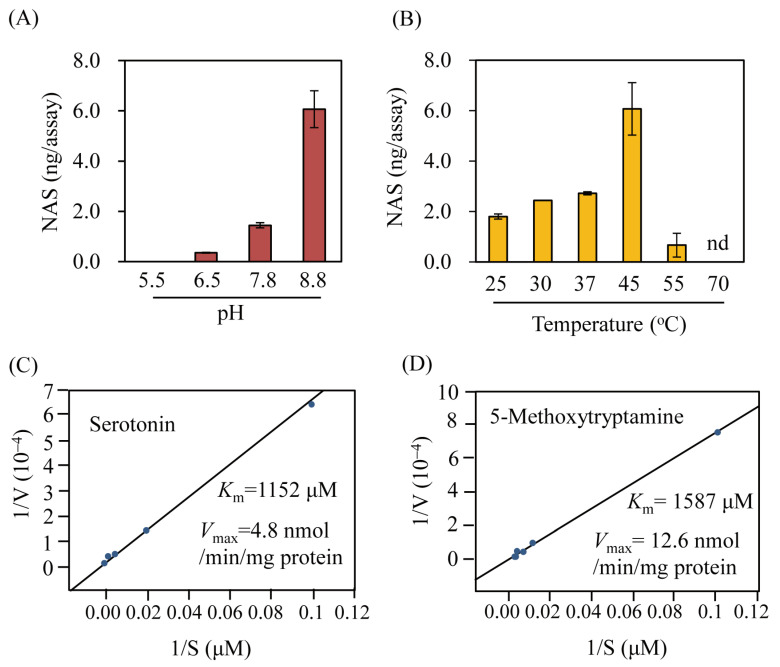
Enzyme kinetics of OsSNAT3. SNAT activity as a function of (**A**) pH and (**B**) temperature, and determination of *K*_m_ and *V*_max_ values of OsSNAT3 using (**C**) serotonin and (**D**) 5-methoxytryptamine (5-MT) as substrates. Recombinant purified OsSNAT3 (1 µg) was assayed in the presence of different serotonin and 5-MT concentrations for 1 h at different temperatures and pH values, followed by high-performance liquid chromatography detection of *N*-acetylserotonin (NAS) and melatonin. Kinetic values of *K*_m_ and *V*_max_ were determined using Lineweaver–Burk plots. Values are presented as the mean ± SD (*n* = 3). nd, not detected.

Two forms of the recombinant OsSNAT3 protein—N-terminal His-tagged OsSNAT3 (His6-OsSNAT3) and C-terminal His-tagged OsSNAT3 (OsSNAT3-His6)—were analyzed for SNAT activity using serotonin as the substrate. As shown in Figure 1D, the SNAT activity of the OsSNAT3-His6 protein was 5-fold higher than that of His6-OsSNAT3, indicating the inhibition of OsSNAT3-mediated catalysis by N-terminal His-tag sequences. The OsSNAT3-His6 recombinant protein was thus employed in further analyses of SNAT enzyme kinetics. Similar to TvSNAT, the optimum SNAT activity of OsSNAT3 was at pH 8.8 (Figure 2A). Peak OsSNAT3 activity was obtained at a temperature of 45 °C with a sharp decrease at 55 °C, in stark contrast to the many other plant SNAT proteins, whose peak activities were at 55 °C (Figure 2B) [29,35]. The *K*_m_ and *V*_max_ values of OsSNAT3 were 1152 μM and 4.8 nmol/min/mg protein, respectively, with serotonin as the substrate (Figure 2C), and 1587 μM and 12.6 nmol/min/mg protein, respectively, with 5-methoxytryptamine as the substrate (Figure 2D). The catalytic efficiency (*V*_max_/*K*_m_) of OsSNAT3 was 2-fold higher when 5-methoxytryptamine rather than serotonin served as the substrate. Based on *K*_m_ values for OsSNAT1 and OsSNAT2 of 270 μM and 371 μM, respectively, with serotonin as the substrate, it is likely that OsSNAT3 is functionally involved in melatonin biosynthesis under conditions of high serotonin levels, such as those induced by senescence and Cd stress [20].

**Figure 3:**
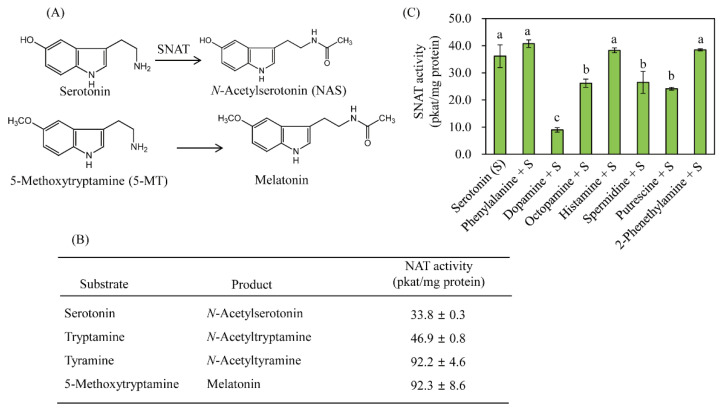
SNAT reactions and substrate preferences. (**A**) SNAT activity toward serotonin and 5-MT substrates. (**B**) SNAT activity toward other substrates. (**C**) Activity of recombinant purified OsSNAT3 toward serotonin (0.5 mM) and various amines (0.5 mM) at 55 °C and pH 8.8. Values are presented as the mean ± SD (*n* = 3). Different letters indicate significant differences vs. the wild type (Tukey’s HSD; *p* < 0.05).

SNAT accepts multiple substrates, including tyramine, tryptamine, and polyamines [36]. As shown in Figure 3, the best substrates for OsSNAT3 were 5-methoxytryptamine (92.3 pkat/mg protein) and tyramine (92.2 pkat/mg protein), followed by tryptamine (46.9 pkat/mg protein) and serotonin (33.8 pkat/mg protein) (Figure 3B). Unlike TvSNAT, the activity of which is lowest when 5-methoxytryptamine is the substrate [21], OsSNAT3 activity peaked in response to this substrate. Both sheep SNAT and yeast SNAT also reach peak enzyme activity when provided with 5-methoxytryptamine [37]. In addition to the aforementioned arylalkylamines, other arylalkylamines (dopamine, octopamine, 2-phenylethylamine, and histamine) and polyamines (spermidine and putrescine) were tested for their acceptance as OsSNAT3 substrates. Due to the absence of respective standards, an SNAT inhibition assay (0.5 mM serotonin) was conducted in the presence of each potential substrate (0.5 mM) to determine its inhibitory effect on SNAT activity. Analogous to TvSNAT, OsSNAT3 activity was strongly inhibited by spermidine and octopamine (Figure 3C) and significantly inhibited by putrescine and dopamine. These data indirectly indicate that OsSNAT3 is able to acetylate dopamine, spermidine, putrescine, and octopamine, yielding *N*-acetyldopamine, *N*-acetylspermidine, *N*-acetylputrescine, and *N*-acetyloctopamine, respectively. These results suggest a broader substrate affinity of OsSNAT3 than TvSNAT. However, the relationship between acetylated polyamines and OsSNAT3, particularly with respect to stress defense responses in rice, remains to be studied in detail.

### 2.3. Subcellular Localization of OsSNAT3

**Figure 4:**
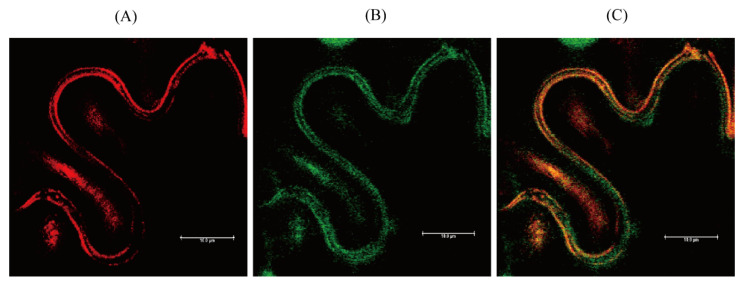
Subcellular localization of OsSNAT3. (**A**) Red fluorescence of OsSNAT3-mCherry. (**B**) Green fluorescence of cytoplasmic green fluorescent protein (GFP). (**C**) Merged fluorescence images (A + B). Thirty-day-old tobacco seedlings infiltrated with *Agrobacterium tumefaciens* (GV2260) containing XVE-inducible *OsSNAT3*-mCherry or constitutive 35S:GFP (cytosolic marker). Bars = 10 μm.

The subcellular localization of OsSNAT3 was determined through in silico analysis. First, the possible presence of transit or signal sequences was examined in a TargetP analysis [38], followed by the use of cNLS Mapper to identify a nuclear localization signal (NLS) [39]. Although neither transit nor signal sequences are predicted by OsSNAT3 polypeptides, cNLS Mapper predicted two putative NLS sequences (LKKLNTALEPVRYNEKYYHDTIASKEFS and DLCEKQNIPEIYLHVQTNNDDAIAFYKKFGFE) with scores of 3.2 and 4.3, implying both nuclear and cytoplasmic subcellular localization. In agreement with the cNLS prediction, an OsSNAT3-mCherry fusion protein was localized to the cytoplasm (Figure 4) when the fusion construct was transiently expressed in leaf epidermal cells of *Nicotiana benthamiana*, a native Australian tobacco species. While the subcellular localization of OsSNAT3 in the cytoplasm was predominantly observed in our experiments, the expression of OsSNAT3 in other subcellular compartments cannot be ruled out because the expression of *Arabidopsis thaliana* Naa50, an ortholog of OsSNAT3, in the nucleus, cytoplasm, and ER has been reported [40]. OsSNAT3 expression thus differs from that of OsSNAT1 and OsSNAT2, which have been localized to chloroplasts [30,31]. The location of SNAT protein isoforms—as key enzymes in melatonin biosynthesis in plants—in the cytoplasm, nucleus, chloroplasts, and mitochondria indicates the ubiquity of cellular melatonin biosynthesis [20,29,41,42].

### 2.4. Reduced Synthesis of Melatonin in OsSNAT3-Suppressed Transgenic Rice Plants

**Figure 5:**
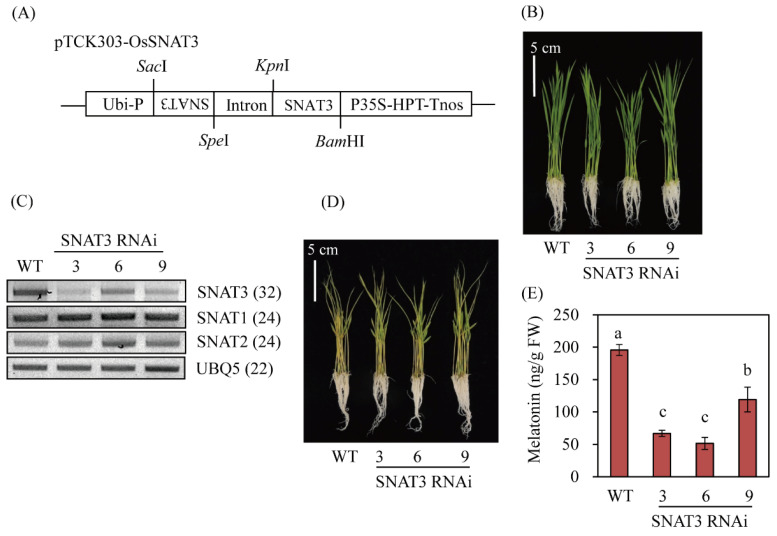
Generation of *OsSNAT3* RNAi transgenic rice and the melatonin content of rice seedlings. (**A**) RNAi binary vector used for *OsSNAT3* suppression. (**B**) Phenotypes of 7-day-old rice seedlings. (**C**) RT-PCR analyses of transgenic and wild-type 7-day-old rice seedlings. (**D**) Photograph of 7-day-old rice seedlings treated for 3 days with 0.5 mM CdCl_2_. (**E**) Melatonin contents of 7-day-old rice seedlings treated for 3 days with 0.5 mM CdCl_2_. *Ubi-P*, maize ubiquitin promoter; *HPT*, hygromycin phosphotransferase; WT, wild type; *UBQ5*, rice ubiquitin 5 gene. GenBank accession numbers of *SNAT1*, *SNAT2*, *SNAT3*, and *UBQ5* are AK059369, AK068156, AK241100, and AK061988, respectively. Different letters indicate significant differences vs. the wild type (Tukey’s HSD; *p* < 0.05).

From 14 T_0_-independent transgenic lines generated via *Agrobacterium*-mediated rice transformation in vitro, 12 yielded T_1_ seeds; the other two lines died during growth under field conditions. The obtained T_1_ seeds, which exhibited hygromycin resistance and a sensitivity ratio of 3:1, were grown and three T_2_ homozygous lines (lines 3, 6, and 9) were selected for further analyses (Figure 5). As shown in Figure 5B, the phenotypes of the 7-day-old *OsSNAT3* RNAi lines and wild-type seedlings did not significantly differ. However, in all three transgenic RNAi lines, *OsSNAT3* transcript levels were much lower than those of the wild type, thus confirming the successful generation of the *OsSNAT3* RNAi lines. *OsSNAT3* suppression did not affect the transcript levels of *OsSNAT1*, whereas those of *OsSNAT2* were slightly elevated, indicative of the feedback regulation between *OsSNAT2* and *OsSNAT3* transcripts. To determine the effect of *OsSNAT3* downregulation on melatonin levels, 7-day-old rice seedlings were challenged with 0.5 mM Cd for 3 days, after which melatonin induction was assessed by HPLC. As shown in Figure 5E, the average rate of melatonin production in wild-type seedlings was 195 ng/g fresh weight (FW) and that of the transgenic *OsSNAT3* RNAi lines was 77 ng/FW, a 2.5-fold difference. This result indicates that *OsSNAT3* mRNA is functionally coupled to in vivo melatonin synthesis in rice plants.

### 2.5. A Decrease in Endogenous Melatonin Aggravates Cd Toxicity

**Figure 6:**
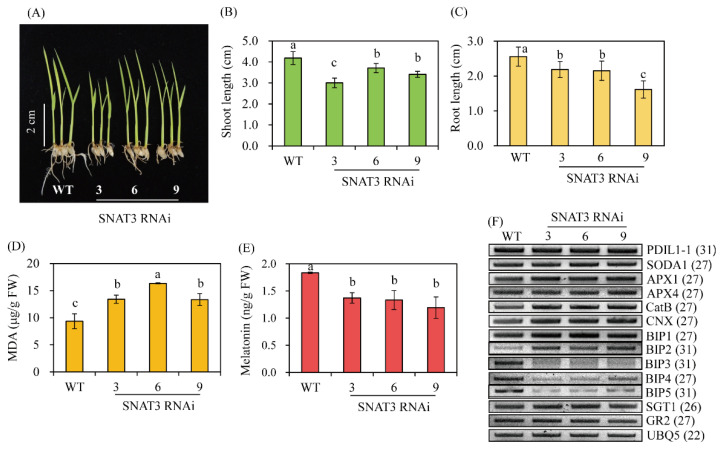
Enhanced Cd stress susceptibility in *OsSNAT3* RNAi transgenic rice plants. (**A**) Growth phenotype, (**B**) shoot length, (**C**) root length, (**D**) malondialdehyde (MDA) contents, and (**E**) melatonin content in 7-day-old rice seedlings. (**F**) Gene expression profiles determined by RT-PCR in Cd-stressed rice plants. Dehusked seeds were surface-sterilized and transferred for 7 days to half-strength Murashige Skoog (MS) medium containing 0.5 mM CdCl_2_ under a 14 h light/10 h dark photoperiod and an incubation temperature of 28 °C/24 °C (day/night). Values are presented as the mean ± SD (*n* = 3). Different letters indicate significant differences vs. the wild type (Tukey’s HSD; *p* < 0.05). GenBank accession numbers: *PDIL1–1* (AK068268), *SODA1* (AAA62657), *APX1* (AB050724), *APX4* (AK104490), *CatB* (AK069446), *CNX* (AK069118), *BiP1* (AK119653), *BiP2* (BAS86012), *BiP3* (BAS93656), *BiP4* (AK106696), *BiP5* (BAF23108), *SGT1* (BAF05534), *GR2* (BAF10399), and *UBQ5* (AK061988).

The enormous induction of endogenous melatonin biosynthesis by Cd in rice has been attributed to melatonin-mediated Cd tolerance [27]. When 7-day-old seedlings were challenged with Cd, as shown in Figure 5D, no significant differences in MDA levels between the wild-type and OsSNAT3 RNAi lines were observed. Thus, the effects of Cd response have been examined directly in MS medium in the presence of Cd (Figure 6). In this study, consistent with the reduced melatonin levels (Figure 6E), seedling growth in the *OsSNAT3* RNAi lines was strongly reduced compared to the wild type when dehusked rice seeds were grown for 7 days in half-strength MS medium containing 0.5 mM CdCl_2_ (Figure 6A–C), with a much shorter root length (Figure 6C). In addition, the levels of malondialdehyde (MDA), a marker of lipid peroxidation and oxidative stress, were higher in *OsSNAT3* RNAi than in wild-type plants (Figure 6D). To identify the major genes responsible for the melatonin-regulated Cd response, we examined the expression of the antioxidant-related genes *SODA1* (encoding Mn-superoxide dismutase), *APX1* (ascorbate peroxidase), *GR2* (glutathione reductase), and *CatB* (catalase), and of the chaperone-related genes *PDIL1–1* (protein disulfide isomerase-like), *CNX* (calnexin), *BiP* (binding protein), and *SGT1* (suppressor of the G2 allele of skp1). In the *OsSNAT3* RNAi lines, among the antioxidant-related genes, only *CatB* expression was changed, with an increase compared to the wild type; there was no change in the expression levels of the other studied enzymes (Figure 6F). Among the chaperone genes, *BiP3*, *BiP4*, and *BiP5* expressions were strongly downregulated while *BiP1*, *BiP2*, and *CNX* expressions were upregulated in the *OsSNAT3* RNAi lines, indicating the differential expression of *BiP* genes. A similar differential expression between *OsSNAT3* RNAi lines and the wild type was not observed for *PDIL1–1* and *SGT1*. These results indicate that *BiP3*, *BiP4*, and *BiP5* expressions are closely associated with the melatonin-mediated Cd susceptibility response.

### 2.6. Increased Endogenous Melatonin Elevates Cd Tolerance in OsSNAT3-Overexpressing Lines

**Figure 7:**
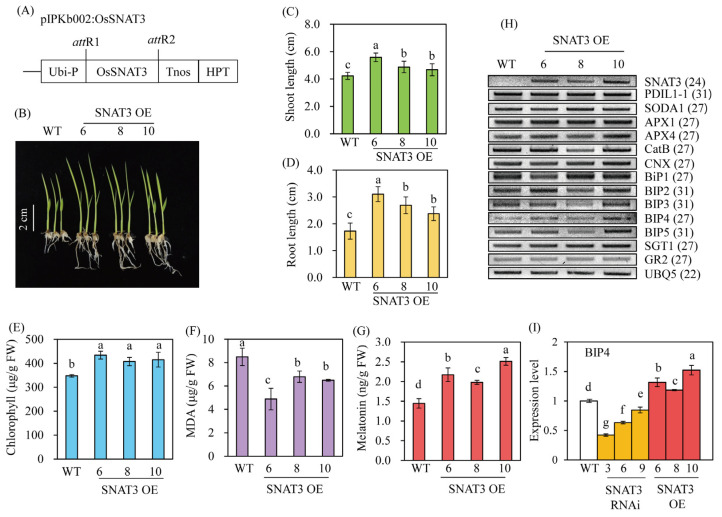
Enhanced Cd tolerance in *OsSNAT3*-overexpressing transgenic rice plants. (**A**) Schematic diagram of the binary vector for *OsSNAT3* overexpression. (**B**) Phenotype of Cd-treated 7-day-old rice seedlings. (**C**) Shoot length, (**D**) root length, and (**E**) chlorophyll, (**F**) MDA, and (**G**) melatonin contents in Cd-treated plants. (**H**) Gene expression profiles of Cd-treated rice plants, as determined by RT-PCR. (**I**) *BiP4* expression levels determined in a quantitative real-time PCR analysis of Cd-stressed rice plants. *OsSNAT3*, *Oryza sativa serotonin N-acetyltrasferase3; Ubi-P*, maize ubiquitin promoter; *HPT*, hygromycin phosphotransferase; WT, wild type; *UBQ5*, rice ubiquitin 5 gene. GenBank accession numbers are listed in Figure 6. Different letters indicate significant differences vs. the wild type (Tukey’s HSD; *p* < 0.05).

To examine the coupling of melatonin-regulated Cd tolerance to *BiP* expression, transgenic rice plants overexpressing *OsSNAT3* (SNAT3-OE) were generated (Figure 7A). From the 14 independent T_0_ transgenic lines, three T_2_ homozygous lines (lines 6, 8, and 10) overexpressing *SNAT3* (SNAT3-OE) were selected (Figure 7H). When SNAT3-OE seeds were grown for 7 days in MS medium containing 0.5 mM Cd, the seedlings exhibited enhanced growth compared to wild-type seedlings (Figure 7B–D). Chlorophyll levels were also higher in SNAT3-OE than in wild-type seedlings (Figure 7E), while MDA levels were reduced. These results indicate that greater melatonin production by the SNAT3-OE lines resulted in their better tolerance of Cd stress than wild-type seedlings (Figure 7G). *BiP4* was identified as the main gene responsible for conferring Cd tolerance in the SNAT3-OE lines, and its expression was accordingly higher than in wild-type plants, as shown by PCR and quantitative real-time PCR analyses (Figure 7H,I). The expressions of *BiP3* and *BiP5* did not differ between SNAT3-OE and wild-type plants.

## 3. Discussion

While many heavy metals, including copper, iron, zinc, cobalt, and manganese, are required as micronutrients for plant growth, Cd is toxic, causing severe damage to all living organisms, including plants and animals [43,44,45]. Among the many adverse effects of Cd on plant growth and development are the inhibition of root growth, leaf chlorosis, a reduction in photosynthesis, the inhibition of nutrient uptake and germination, and yield reduction [45]. To cope with Cd stress, plants have evolved a number of defense mechanisms, such as the chelation of Cd by metallothionein and phytochelatins, the regulation of heavy metal transporters, the induction of enzymatic and nonenzymatic antioxidants, and increased levels of plant hormones and HSPs [2,5,45].

Melatonin is a pleiotropic molecule found in all living organisms examined so far. It acts as a neurohormone in animals but as a signaling molecule in plants, although its potent antioxidant activity is common in all organisms [46,47]. Its antioxidant scavenging activity is directed against a diverse array of oxidants, including ROS and reactive nitrogen species, but as a signaling molecule melatonin also induces cellular antioxidant enzymes, such as catalase, APX1, and SOD [19,24,48]. Melatonin thus orchestrates the response to both biotic and abiotic stresses, including Cd stress in plants [25,27,49]. For instance, in rice seedlings treated with 1 µM melatonin in a hydroponic nutrient solution, Cd accumulation is efficiently alleviated by a mechanism involving enhanced hemicellulose levels in conjunction with decreased levels of transporter genes, thus lowering Cd intake [50]. In pepper seedlings, treatment with 5 µM melatonin enhances Cd stress tolerance by lowering leaf/root Cd concentrations and ROS contents while upregulating antioxidant genes encoding *SOD* and *APX* [7]. In *Wolffia arrhiza* exposed to 25 µM melatonin, Cd detoxification is increased via elevated phytochelatin and photosynthetic pigments [51]. In tomato seedlings exposed to Cd, melatonin treatment enhances the content of ascorbic acid and glutathione [52]. In Xing et al. [53], melatonin alleviated Cd-induced oxidative stress in tomato plants by increasing the ratio of reduced GSH to oxidized GSH and that of ascorbic acid to dehydroascorbic acid, in addition to inducing phytochelatin levels.

Rice is a major dietary crop capable of high levels of Cd adsorption [50], which together with its relatively high levels of melatonin production makes it well suited for the study of melatonin biosynthesis and Cd tolerance in plants [20]. Previous studies have shown that melatonin production is dramatically induced in Cd-treated rice plants [20,27,54]. Our findings of endogenous melatonin-mediated Cd tolerance by *BiP4* induction in rice, together with the studies summarized above, demonstrate the ability of melatonin to mitigate the negative effects of Cd stress through several different defense mechanisms. Therefore, melatonin is a pleiotropic molecule whose functions include protection against Cd stress in plants.

Heavy metal stresses inhibit cellular protein homeostasis by interfering with protein folding, resulting in the dysfunction of essential enzymes and other proteins. To maintain optimum protein homeostasis in the presence of heavy metal stress, plants induce the expression of HSPs, which assist in protein folding, prevent protein aggregation, and accelerate the degradation of aberrant proteins [8]. BiPs are HSP70 proteins residing in the ER [8]. They include *BiPD*, involved in drought tolerance in soybean and tobacco [55]; *BiP3*, associated with pathogen resistance in rice [56]; and *BiP1* and *BiP4*, involved in seed storage protein regulation [15], osmotic stress tolerance [27], and ER stress tolerance [13,27] in rice. However, while HSPs are well known to ameliorate the stress response in various plant species, endogenous melatonin-mediated *BiP4* expression in response to Cd stress has not been examined in plants.

**Figure 8:**
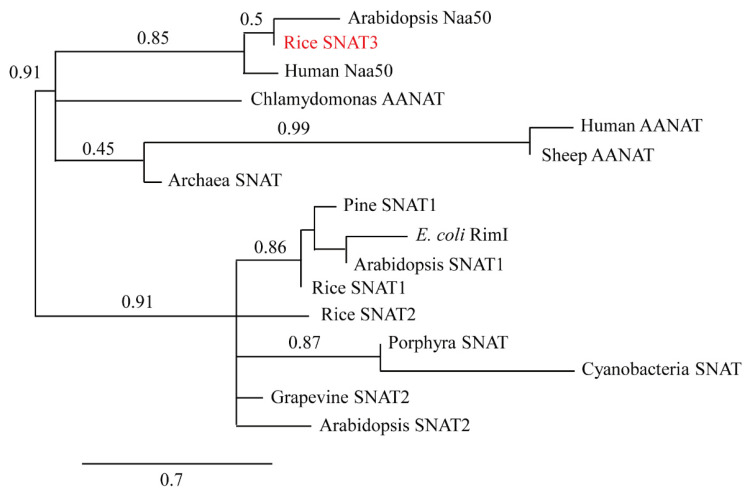
Phylogenetic tree of rice SNAT3 (OsSNAT3) shown as red words, an archaeal SNAT ortholog. The scale bar represents 0.3 substitutions per site. GenBank accession numbers are as follows: *Arabidopsis* Naa50 (NM_121172), rice SNAT3 (AK241100), human Naa50 (BAB14397), Chlamydomonas arylalkylamine *N*-acetyltransferase (AANAT) (AB474787), human AANAT (NP_001079), sheep AANAT (NP_001009461), archaea SNAT (NC_002689), pine SNAT1 (PSY00020345), *E*. *coli* RimI (WP_137442509), *Arabidopsis* SNAT1 (At1g32070), rice SNAT1 (AK059369), rice SNAT2 (AK068156), porphyra SNAT (NC_007932), cyanobacteria SNAT (NP_442603), grapevine SNAT2 (RVX06207), and *Arabidopsis* SNAT2 (At1g26220).

SNAT is the penultimate or last enzyme for melatonin biosynthesis in animals and plants, catalyzing serotonin and 5-methoxytryptamine to yield *N*-acetylserotonin and melatonin, respectively [20]. Animals and humans possess a single copy of SNAT, but plants, including rice, harbor at least three isogenes, *SNAT1*, *SNAT2*, and *SNAT3*, with low amino acid identity among them. All three *SNAT* genes are functionally involved in melatonin synthesis, but their subcellular locations and physiological functions vary. Thus, both SNAT1 and SNAT2 localize in chloroplasts [29], while SNAT3 is found in the cytoplasm. *SNAT2*, but not *SNAT1*, is associated with brassinosteroid synthesis [57]. Importantly, *SNAT1* and *SNAT2* are specific to plants, whereas *SNAT3* orthologs are universally present in a diverse array of organisms, including rice (this report), archaea [21], humans [35], and *Escherichia coli* [22] (Figure 8). Our study identified rice *SNAT3* as a functional ortholog of archaeal *SNAT*. Its overexpression and downregulation were closely coupled with melatonin synthesis and the Cd stress response, with the latter involving the upregulated expression of the *BiP4* chaperone gene. Thus, it is tempting to speculate that melatonin-based Cd tolerance is one of many defense mechanisms by which rice and possibly other plants cope with Cd stress, with the induction of Cd intake-related genes [50], ROS detoxification genes [7,58], phytochelatin synthesis genes [53], and HSPs [8,27] among the others.

Our findings open a new window to crop improvement in areas with a high soil Cd content, by way of either classical breeding or genetic engineering strategies that increase melatonin synthesis.

## 4. Materials and Methods

### 4.1. Sequence Alignment and Phylogenetic Analysis

Full-length rice *SNAT3* cDNA (GenBank accession number AK241100) was kindly provided by the National Institute of Agrobiological Sciences [31,59,60]. The analysis of amino acid sequence homology was performed with the BLASTp tool (version 2.15.0) using the non-redundant protein sequences databases at the National Center for Biotechnology Information (http://www.ncbi.nlm.nih.gov/, accessed on 18 July 2019). The acetyl coenzyme A binding pocket was computed by the Conserved Domain Database (CDD), which is included in the National Center for Biotechnology Information (NCBL)’s online search services (accessed on 13 August 2019) [61]. We employed BLAST-Explorer [62] for phylogenetic trees analysis (accessed on 29 December 2022). TargetP analysis was used for the prediction of possible transit or signal sequences [38]. To predict the existence of nuclear localization signals (NLSs), the cNLS (calculating NLS scores) Mapper service was employed [39].

### 4.2. Escherichia coli Expression and Purification of Recombinant OsSNAT3 Protein

Two types of *Escherichia coli* expression vectors were used to express the full-length *OsSNAT3*. These two vectors were pET300 (Invitrogen, Carlsbad, CA, USA) and pET28b (Novagen, San Diego, CA, USA), which are designed to express *OsSNAT3* in either N-terminal- or C-terminal-hexahistidine tagged form. As for the pET300 vector, full-length *OsSNAT3* cDNA was amplified by PCR by using a primer set (*OsSNAT3* forward primer, 5′-AAA AAG CAG GCT CCA TGG GCG CCG GGG AAG-3′; *OsSNAT3* reverse primer, 5′-AGA AAG CTG GGT TCA TTT CTT TGT AGC-3′) with a template plasmid containing *OsSNAT3* cDNA provided by the National Institute of Agrobiological Sciences. The first PCR product was used for the template of the second PCR using the *attB* primer set, as described previously [22]. The second *OsSNAT3* PCR product was cloned using gateway recombination reactions in the pDONR221 vector (Invitrogen, Carlsbad, CA, USA) to generate pDONR221-OsSNAT3 plasmid, and then recombined into the destination vector pET300/NT-DEST (Invitrogen) resulting in the pET300-OsSNAT3 plasmid, according to the manufacturer’s procedure. As for the pET28b vector, the full-length *OsSNAT3* was amplified by PCR with *Nco*I forward primer (5′-ACC ATG GGC GCC GGG GAA GGG-3′) and *Xho*I reverse primer (5′-CTC GAG TTT CTT TGT AGC AGC CTG ACC-3′). The resulting *OsSNAT3* PCR product was first cloned into a TA cloning vector (RBC Bioscience, New Taipei City, Taiwan) followed by *Nco*I and *Xho*I digestion. The *Nco*I and *Xho*I inserts of *OsSNAT3* were then ligated into the same restriction endonuclease sites of the pET28b vector. Both pET300-OsSNAT3 and pET28b-OsSNAT3 plasmids were transformed into *E. coli* BL21 (DE3) strains (Invitrogen) using the heat shock method. Bacterial culture and recombinant protein purification procedures were performed according to the manufacturer’s recommendations (Qiagen, Tokyo, Japan).

### 4.3. Measurement of SNAT Enzyme Kinetics

The purified recombinant OsSNAT3 protein (0.5 μg) was assayed in the presence of 0.5 mM acetyl-CoA and varying concentrations of serotonin (or other substrates) in 100 mM potassium phosphate (pH 8.8 or varying pH) at 45 °C (or other temperatures) for 30 min. The in vitro enzymatic reaction products and endogenous melatonin contents in the transgenic rice seedlings were quantified by high-performance liquid chromatography (HPLC), as described previously [21]. Lineweaver–Burk plots were generated for calculating substrate affinity (*K*_m_) and the maximum reaction rate (*V*_max_) with the OsSNAT3 recombinant protein (0.25 μg) after 20 mins of enzymatic reaction. Protein levels were determined using the Bradford method and a protein assay dye (Bio-Rad, Hercules, CA, USA). The analysis was performed in triplicate.

### 4.4. Subcellular Localization of OsSNAT3

The pER-mCherry binary vector was generously provided by Dr. H.G. Kang (Texas State University, San Marcos, TX, USA). The full-length of *OsSNAT3* cDNA was amplified by PCR using a primer set containing *Asc*I sites (*Asc*I forward primer 5′-GGC GCG CCA TGG GCG CCG GGG AAG GGG AT-3′; *Asc*I reverse primer, 5′-GGC GCG CCG TTT CTT TGT AGC AGC CTG-3′) with a template plasmid cDNA (GenBank accession number AK241100). The resulting *OsSNAT3* PCR product was initially ligated into the TA vector (RBC Bioscience) followed by *Asc*I restriction endonuclease enzyme digestion. The purified *Asc*I insert of *OsSNAT3* was then introduced into the *Asc*I site of the binary vector pER8-mCherry containing the estrogen-inducible XVE promoter (Pxve), resulting in pER8-OsSNAT3-mCherry. The pER8-OsSNAT3-mCherry plasmid construct was transformed into the *Agrobacterium tumefaciens* GV2260 strain using the freeze–thaw method. The *Agrobacterium*-mediated transient expression of OsSNAT3-mCherry fusion protein was identified and confocal microscope analysis (TCS-SP5; Leica, Wetzlar, Germany) was performed, as described previously [30].

### 4.5. Transgenic Rice Plants either Downregulating or Overexpressing OsSNAT3

The pTCK303 RNAi binary vector was utilized to knockdown the expression of the rice Os*SNAT3* gene, as previously described [57]. In brief, a 290-bp *OsSNAT* 3 cDNA fragment positioned in the middle region of the *SNAT3* cDNA was amplified by polymerase chain reaction (PCR) with the following primer set: *OsSNAT3* forward 5′-ACT AGT AAC ACG GCG CTC TTC CCC GTC-3′ (*Spe*I site underlined) and *OsSNAT3* reverse 5′-GAG CTC AGC AAT GGC ATC ATC GTT GTT-3′ (*Sac*I site underlined). The amplified *OsSNAT3* PCR product was first cloned into the T&A cloning vector (T&A: OsSNAT3; RBC Bioscience), from which the Os*SNAT3* insert (antisense) was acquired by the digestion of *Sac*I and *Spe*I restriction enzymes, while the sense *OsSNAT3* insert was obtained by *Kpn*I and *Bam*HI digestion. The antisense *OsSNAT3* was first ligated into the pTCK303 vector, which was predigested by the same restriction enzymes. Thereafter, the sense fragment of the *OsSNAT*3 insert was further ligated into the pTCK303 vector harboring the antisense DNA fragment. The resulting pTCK303:OsSNAT3 RNAi binary vector was transformed into *Agrobacterium tumefaciens* strain LBA4404, followed by rice transformation to the Korean Japonica cultivar Dongjin. The transgenic rice plants were regenerated from calli in the presence of hygromycin via a somatic embryogenesis process, as previously described [57]. As for the *OsSNAT3* overexpression vector construct, the pIPKb002 binary vector [63], which is designed to overexpress the transgene *OsSNAT3* under the control of maize ubiquitin promoter, was used. The pDONR221-OsSNAT3 plasmid was recombined with the pIPKb002 destination vector using the LR clonase enzyme (Invitrogen) to yield the pIPKb002-OsSNAT3 binary plasmid. The pIPKb002-OsSNAT3 binary vector was transformed into *Agrobacterium tumefaciens* LBA4404 and rice, as described above.

### 4.6. RNA Extraction and Reverse Transcription–Polymerase Chain Reaction (RT-PCR) Analysis

Total RNA from rice seedlings was isolated using a NucleoSpin RNA Plant Kit (Macherey-Nagel, Düren, Germany). First-strand cDNA was synthesized from 2 µg of total RNA using EcoDry^TM^ Premix (Takara Bio USA, Inc., Mountain View, CA, USA). The conditions of RT-PCR were begun with initial denaturation at 95 °C (3 min) followed by varying cycles of denaturation at 95 °C (30 s), annealing at 56 °C (30 s), and extension at 72 °C (30 s) in 30 µL of master mix (Takara Bio Inc., Kusatsu, Shiga, Japan). The primer sequences for *OsSNAT3* were forward primer (5′-ATG GGC GCC GGG GAA GGG GAT-3′) and reverse primer (5′-TTT CTT TGT AGC AGC CTG-3′). The other primer sequences were as described in previous reports [14,22,40]. Quantitative real-time PCR was carried out as described previously [35].

### 4.7. Cadmium Treatment and Melatonin Measurement

Dehusked rice seeds were sterilized with 2% NaOCl for 50 min, after which they were thoroughly rinsed with sterile distilled water and sown on half-strength Murashige and Skoog (MS) medium under cool daylight fluorescent lamps (60 μmol m^–2^ s^–1^) (Philips, Amsterdam, The Netherlands) under a 14 h light/10 h dark photoperiod at 28 °C/24 °C (day/night). The 7-day-old seedlings collected from MS medium were incubated in 50 mL polypropylene conical tubes containing 30 mL water and 0.5 mM CdCl_2_ (Sigma-Aldrich, St. Louis, MO, USA) and incubated for 7 days for melatonin quantification. As for the cadmium response experiment, the surface-sterilized rice seeds were sown and grown on half-strength MS medium containing a 0.5 mM concentration of cadmium for 7 days, as described above. Melatonin contents were measured from frozen samples (0.1 g), which were pulverized to a powder in liquid nitrogen using the TissueLyser II (Qiagen, Tokyo, Japan). The sample powders were then extracted with 1 mL chloroform followed by centrifugation for 10 min at 12,000 rpm, and then the supernatants (200 µL) were evaporated and dissolved in 0.1 mL of 40% methanol. The resulting 10 µL aliquots were subjected to high-performance liquid chromatography (HPLC) using a fluorescence detector system (Waters, Milford, MA, USA), as described previously [30].

### 4.8. Measurements of Chlorophyll and Malondialdehyde

The powder of the rice seedlings (100 mg) was extracted with 1 mL of 0.1 mM NH_4_OH (containing 80% acetone). Chlorophyll concentrations were determined at wavelengths of 647, 644, and 750 nm using a spectrophotometer (MicroDigital Nabi, GyungGi, Republic of Korea) according to Porra et al. [64]. As for measuring the malondialdehyde (MDA) levels, the powder (50 mg) was extracted with 1.5 mL of reaction buffer containing 0.5% thiobarbituric acid and 20% trichloroacetic acid. The supernatants decanted from centrifugation at 12,000× *g* for 15 min were boiled at 95 °C for 25 min and placed on ice for 5 min. MDA content was recorded at wavelengths of 440, 532, and 600 nm using a spectrophotometer (MicroDigital Nabi) with a molar extinction coefficient of 156/nmol/L/cm.

### 4.9. Statistical Analysis

The data were evaluated by analysis of variance using IBM SPSS Statistics 25 software (IBM Corp. Armonk, NY, USA). Different letters above the histograms indicate significantly different values at *p* < 0.05 according to Tukey’s post hoc honestly significant difference (HSD) test. Data are presented as means ± standard deviations.

## 5. Conclusions

Elucidating Cd tolerance mechanisms in plants is the first step toward generating Cd-tolerant crops by either classical breeding or genetic engineering strategies. SNAT is the rate-limiting enzyme for melatonin biosynthesis in plants and animals. This report describes the cloning and characterization of the *SNAT3* gene from rice, a functional ortholog of archaeal *SNAT*. The downregulation of *SNAT3* resulted in reduced melatonin synthesis and an enhanced susceptibility to Cd stress, whereas its overexpression increased melatonin synthesis and improved Cd tolerance through a mechanism involving the BiP4 chaperone, an ER-resident HSP, although conclusions about the relationship between OsSNAT3 and BIP4 need more evidence. These results suggest that the adoption by the agriculture industry of melatonin engineering and/or exogenous melatonin application could allow crops to withstand Cd stress, thus improving yields and allowing the harvesting of safe food in Cd-contaminated fields.

## Data Availability

Data are contained within the article.
